# Hierarchical visible-infrared-microwave scattering surfaces for multispectral camouflage

**DOI:** 10.1515/nanoph-2022-0254

**Published:** 2022-07-14

**Authors:** Yun Huang, Yining Zhu, Bing Qin, Yiwei Zhou, Rui Qin, Pintu Ghosh, Min Qiu, Qiang Li

**Affiliations:** State Key Laboratory of Modern Optical Instrumentation, College of Optical Science and Engineering, Zhejiang University, Hangzhou 310024, China; Key Laboratory of 3D Micro/Nano Fabrication and Characterization of Zhejiang Province, School of Engineering, Westlake University, Hangzhou 310024, China; Institute of Advanced Technology, Westlake Institute for Advanced Study, Hangzhou 310024, China

**Keywords:** hierarchical, Lambertian surface, low-emissivity, multispectral camouflage, scattering

## Abstract

Multispectral camouflage, especially for the infrared-microwave range, is an essential technology for the safety of facilities, vehicles, and humans. So far, it has been realized mainly by high infrared specular reflection and high microwave absorption. However, external infrared sources can expose the target through specular reflection; also, the heat production from microwave absorption can increase the infrared radiation. This work proposes a multispectral camouflage scheme based on hierarchical visible-infrared-microwave scattering surfaces to address these issues. The proposed device exhibits: (1) low infrared emissivity (*ε*_8–14 μm_ = 0.17) and low infrared specular reflectivity (*R*_*s*__8–14 μm_ = 0.13), maintaining low infrared radiation and capability to overcome the presence of an external infrared source simultaneously; (2) high scattering in microwave range, with −10 dB radar cross section reduction bandwidth of 8–13 GHz, simultaneously achieving microwave camouflage and reducing the heat production; (3) tunability of color for visible camouflage. This work proposes a method to control scattering over visible-infrared-microwave bands, thereby introducing a new design paradigm for modern camouflage technology.

## 1 Introduction

Camouflage technology [1, 2], which aims at blending an object into its surroundings, is essential in modern systems for protecting vital facilities, vehicles, and humans. With the combination of detecting methods operating in different spectrum ranges, it becomes imperative to improve the compatibility of camouflage technology. Therefore, the development of multispectral camouflage technology is attracting a lot of attention of leading researchers in recent times. Camouflage technologies in microwave (MW), infrared (IR), and visible (VIS) wavelength ranges have different requirements on material, structure and design paradigms. For MW camouflage, the critical point is to reduce the reflected signal of radar. There are two ways to achieve MW camouflage, i.e., either absorbing the incident microwave through absorbent materials [3] and absorbent structures [4] (which is also the same mechanism for the EMI shielding, e.g., by using epsilon-near-zero (ENZ) materials [5, 6] and electromagnetic treatment [7–9]) or scattering the incident microwave through different methods, including artificial magnetic conductor [10], frequency selective surfaces [11], and metasurfaces [12]. IR camouflage, especially for the long wavelength infrared (LWIR) band (8–14 μm) – the most used IR detection band, usually aims at reducing the IR radiation from the target to match that from the surrounding environment. It has been realized by tuning the target’s emissivity through various approaches, including paints [13, 14], metal-based surfaces [15, 16], graphene [17, 18], phase change materials [19, 20], epsilon-near-zero (ENZ) materials [21], photonic crystals [22–26], plasmonic structures [27–29], metasurfaces [30, 31], metamaterials [32, 33], and low-emissivity Lambertian surfaces [34] or by tuning its temperature through different thermal regulation techniques, including thermal insulation [35], Peltier plates [36], and transformation thermotics [37–40]. For visible camouflage, the modification of reflectivity is the key so that the color of the target matches the surrounding background, which can be achieved by using pigments [41], photonic crystals [42], metasurfaces [43], optical films [44], etc.

**Figure 1:**
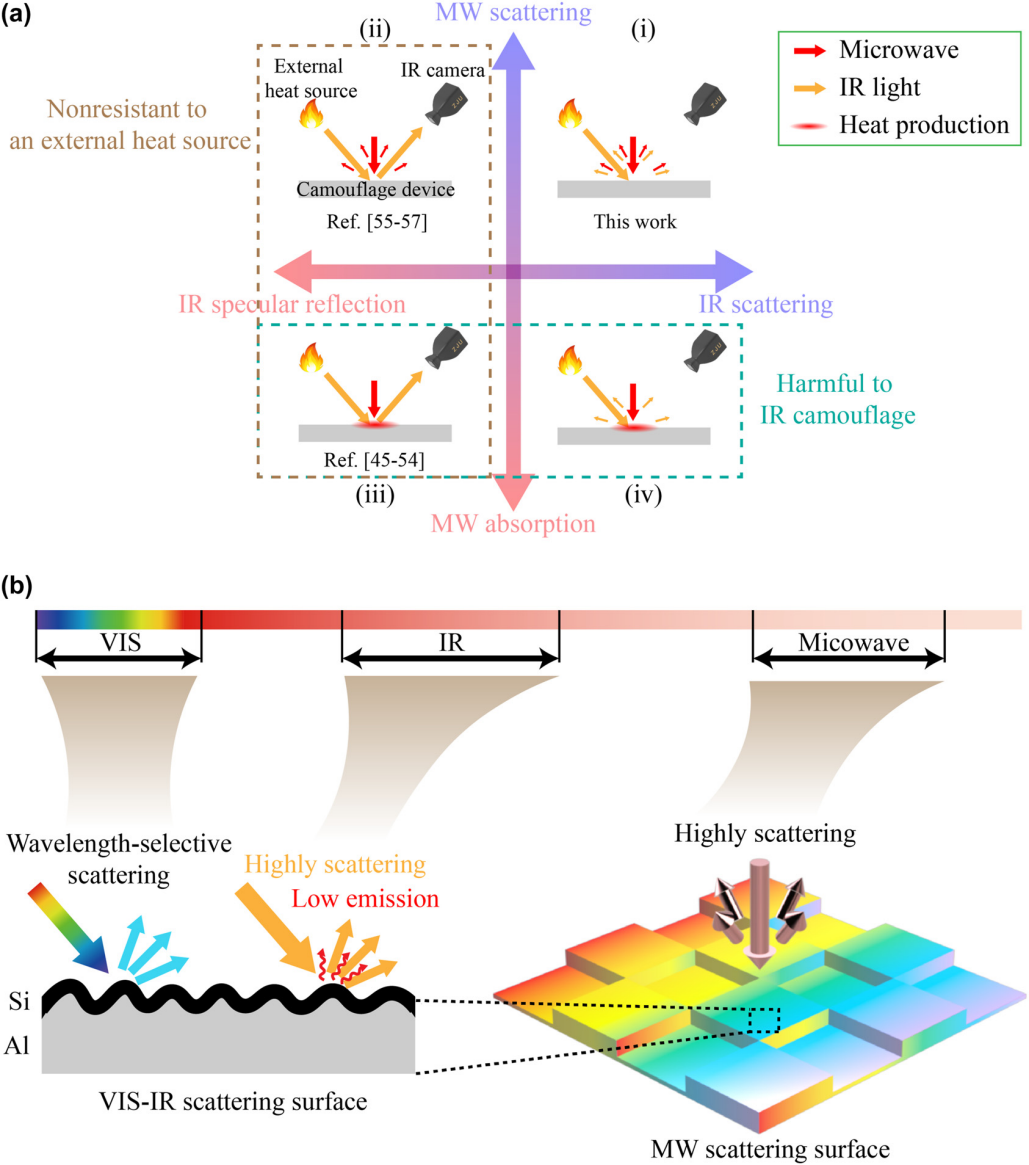
Hierarchical VIS-IR-MW scattering surface for multispsctral camouflage. (a) Comparison of different IR-MW camouflage schemes. (b) Schematic of the hierarchical VIS-IR-MW scattering surface.

IR-MW camouflage can be divided into four categories in terms of the properties in the IR and MW wavelength ranges (Figure 1(a)). IR-specular-reflection-and-MW-absorption camouflage (panel (iii) in Figure 1(a)) has been realized by following diverse methods, including hierarchical metamaterials [45–47], frequency-selective-surfaces-based MW absorbers [48–50], and metasurfaces [51–54]. However, this design not only suffers from the heat production by microwave absorption (which weakens the IR camouflage performance), but also gets exposed to an IR camera by reflecting the IR radiation of external heat sources (i.e., it cannot overcome the influence of external heat sources). IR-specular-reflection-and-MW-scattering camouflage (panel (ii) in Figure 1(a)) is usually realized by wavefront modulation through arrays with different reflection phases [55–57]. This design can eliminate heat production by microwave absorption, but it still fails to eliminate the influence of external heat sources. IR-MW-scattering camouflage (panel (i) in Figure 1(a)) can simultaneously resist the influence of external heat sources and reduce the heat production from the microwave absorption; however, this design has so far slipped out of the multispectral camouflage schemes at status quo. Due to the distinguished difference in size, VIS camouflage can be easily integrated with the IR-MW camouflage techniques mentioned above.

This work demonstrates multispectral camouflage based on hierarchical VIS-IR-MW scattering surfaces. The scheme of multispectral scattering camouflage in this work presents several distinct advantages: (1) highly scattering in MW range – with −10 dB radar cross section reduction (RCSR) bandwidth of 8–13 GHz (covering the whole X band), it simultaneously achieves MW camouflage and reduces heat production; (2) low IR emissivity (average 0.17 over 8–14 μm) and low IR specular reflectivity (average 0.13 over 8–14 μm) – it shows excellent resistance to an external heat source (100 °C) in the IR camouflage, with a 51% (39%) reduction of radiation intensity of the reflected thermal image when the target temperature is 25 °C (100 °C) compared to a smooth device; (3) tunable color (e.g., brown, yellow, purple, etc.) for VIS camouflage.

## 2 Methods

***Calculation of spectrum of the layered structure***: Transfer-matrix method (TMM) is used to calculate the spectrum of the Si–Al layered structure (Section 1 of Supplementary Material).

***Calculation of color gamut***: Based on the calculated visible reflection spectrum, the color gamut is calculated under AM1.5 solar irradiance spectra and the CIE1931 color space (Section 2 of Supplementary Material).

***Simulation of MW property***: Commercial software Lumerical FDTD is used to simulate the reflection phase difference and RCSR of the checkerboard structure (Section 3 of Supplementary Material).

**Figure 2:**
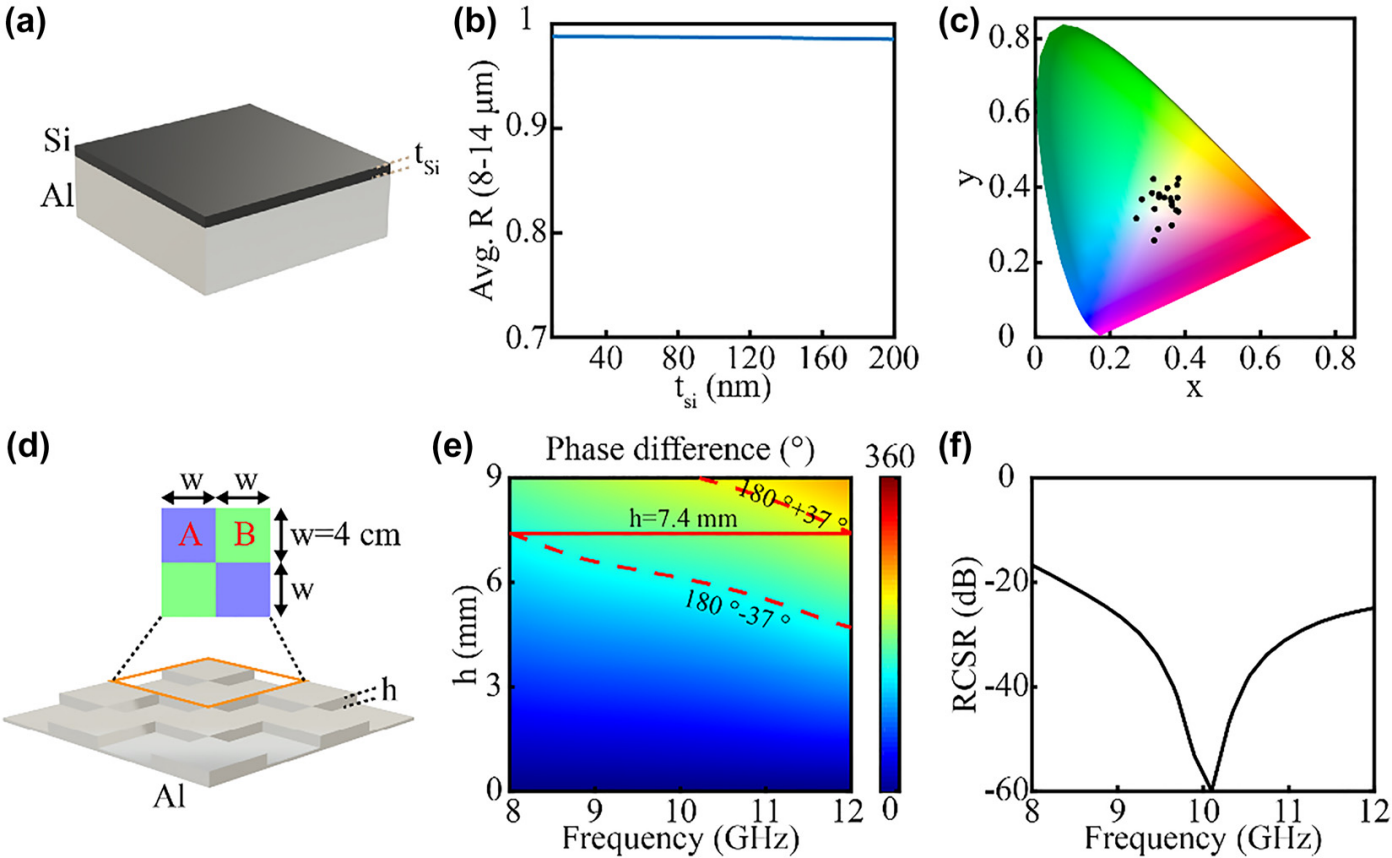
Calculated results for the hierarchical VIS-IR-MW scattering surface. (a) The Si–Al layered structure on the hierarchical VIS-IR-MW scattering surface. (b) Changing the thickness of the Si layer (*t*_si_) from 10 to 200 nm hardly changes the average IR reflectivity (over 8–14 μm) of the Si–Al layered structure – average reflectivity maintained near 1. (c) Calculated color gamut for the Si–Al layered structures (*t*_si_: 10–200 nm). (d) The schematic of the designed MW scattering surface, consisting of Al squares at different heights *h* (the width of each unit is 4 cm). (e) The calculated reflection phase difference between unit A and unit B in (d). The dashed lines present the contour of 180° ± 37°. The solid line illustrates the phase difference for *h* of 7.4 mm. (f) The simulated RCSR of the MW scattering surface with *h* set as 7.4 mm.

***Fabrication of samples***: At first, the low-emissivity smooth checkerboard is fabricated by bonding smooth Al squares (4 × 4 × 7.4 mm) onto a smooth Al plate (16 × 16 cm) arranged as Figure 2(d). Next, Al (100 nm) and Si (of different thicknesses) are deposited onto sandpapers by magnetron sputtering (Denton Discovery 635) to fabricate the VIS-IR scattering surfaces. Finally, the VIS-IR scattering surfaces are stuck onto the low-emissivity smooth checkerboard to form the hierarchical VIS-IR-MW scattering surface.

***Measurement of the morphology of the VIS-IR scattering surface***: The morphology of the VIS-IR scattering surface is measured by a white light optical profiler (NT9100).

***Measurement of IR spectrum***: Fourier transform infrared spectroscopy (FTIR Vertex 70) is used to measure the samples’ IR emissivity and the IR specular reflectivity. For IR emissivity measurements, the samples and a piece of black tape (used as blackbody for reference) are heated to 100 °C to measure the emission intensity. Then the emission intensity of the black tape is used as a reference to calculate the IR emissivity of each sample. For IR specular reflectivity measurements, the IR source inside the FTIR instrument is utilized and reflection is measured by putting the samples and a piece of gold plate (as a reference) under the microscope of the instrument, respectively.

**Figure 3:**
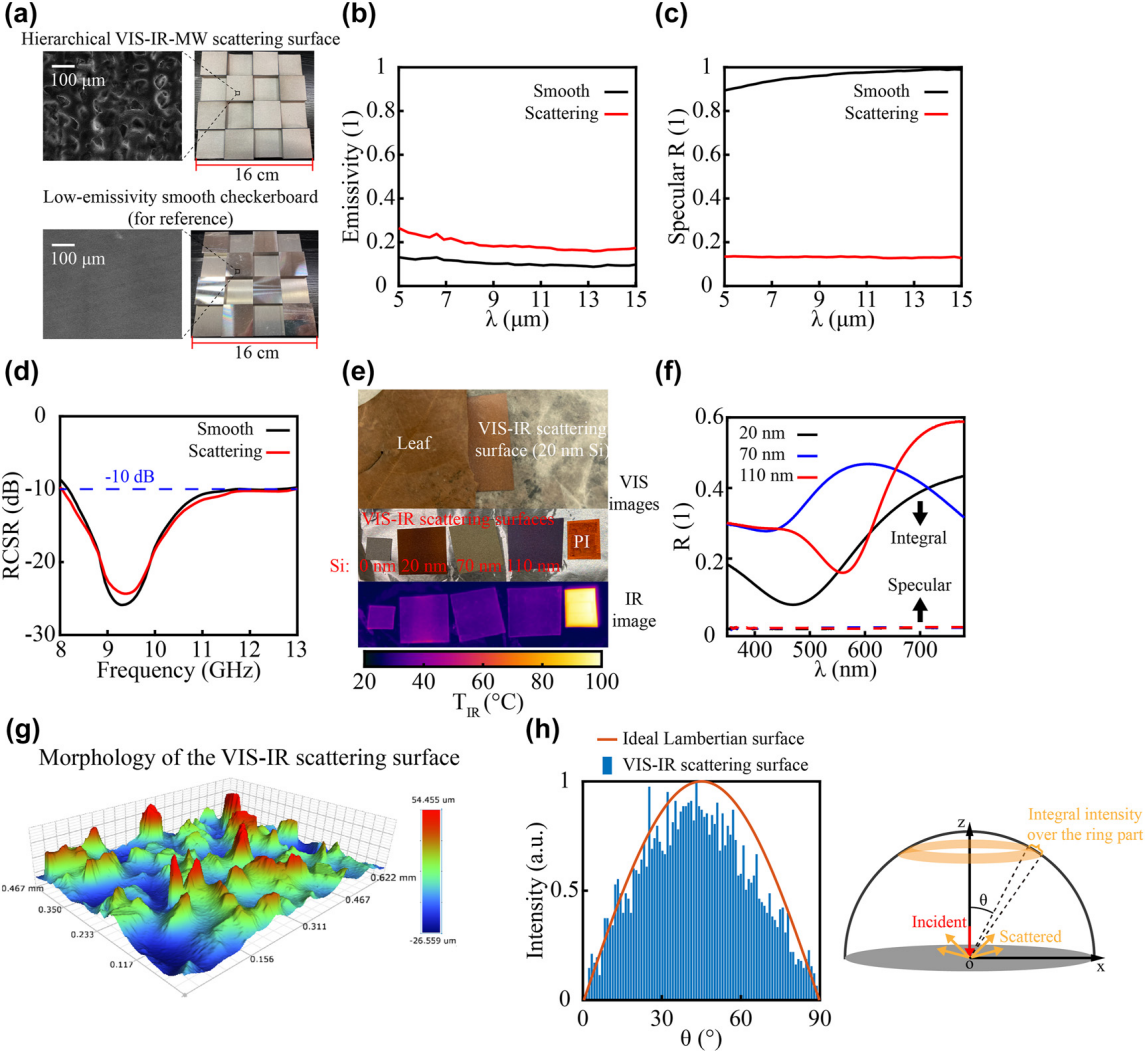
Characterization of the fabricated samples. (a) The SEM images and visible images of the hierarchical VIS-IR-MW scattering surface (upper) and the low-emissivity smooth checkerboard (down). The measured (b) IR emissivity, (c) IR specular reflectivity and (d) RCSR of the hierarchical VIS-IR-MW scattering surface (red) and the low-emissivity smooth checkerboard (black). (e) Top panel: The VIS-IR scattering surface with Si layer of 20 nm mimics the appearance of a leaf. Middle panel: Different appearances of VIS-IR scattering surfaces with Si of 0, 20, 70, and 110 nm. Bottom panel: Apparent temperature of samples in the middle panel at a heating temperature of 100 °C (with highly emissive PI film for reference). (f) Measured VIS integral reflectivity (solid line) and VIS specular reflectivity (dashed line) of the samples in (e) (black: 20 nm Si, blue: 70 nm Si, red: 110 nm Si). (g) Measured morphology of the VIS-IR scattering surface. (h) VIS-IR scattering intensity (integral in the orange ring part, right panel) versus the altitude angel *θ* of the VIS-IR scattering surface (blue bar, statistical calculation results based on the morphology data in (g)) and ideal Lambertian surface (orange line). For the statistical calculation results, the scattering intensity along each *θ* is counted from 0° to 89°, with interval of 1°.

***Measurement of visible spectrum***: The visible reflection spectra of the VIS-IR scattering surfaces (Figure 3(f)) are measured using a universal measurement spectrophotometer (Agilent, Cary7000). For specular reflectivity, the power of the incident light source is measured as reference. For integral reflectivity, an integrating sphere is used in the measurement with the reflection of a standard white plate as reference.

***Measurement of RCSR***: Vector Network Analyzer (Agilent, 8722C) is used to measure the RCSR of the hierarchical VIS-IR-MW scattering surface and the low-emissivity smooth checkerboard, with a smooth Al plate (16 × 16 cm) as a reference.

***IR performance experiment***: An IR camera (Blackbird precision sl, Jenoptik, 8–14 μm), which is placed at a distance less than 1 m from the samples, is used to observe the apparent temperatures of the hierarchical VIS-IR-MW scattering surface, the low-emissivity smooth checkerboard, and the blackbody (PI film). A heating plate is used to heat the devices so as to mimic camouflaging targets with different temperatures. Another heating plate (covered with PI film) is hanged in the air as the external heat source.

## 3 Results and discussion

### 3.1 Design of the hierarchical VIS-IR-MW scattering surface

The hierarchical VIS-IR-MW scattering surface in this design consists of a VIS-IR scattering surface assembled onto an MW scattering surface, where the effective unit size of the VIS-IR scattering surface is several orders smaller than that of the MW scattering surface (Figure 1(b)). The VIS-IR scattering surface serves to maintain low IR radiation, scatter the incident IR light, and tune the appearance color. The MW scattering surface aims to scatter the incident microwave.

*Design of the VIS-IR scattering surface*: The VIS-IR scattering surface in the hierarchical design is a low-emissivity Lambertian surface featuring Si–Al layered structure on a rough substrate. The Si–Al layered structure (Figure 2(a)) is utilized to tune the visible spectrum and keep low IR emissivity. Calculated results show that the Si–Al layered structure can keep the average IR reflectivity (8–14 μm) near 1 when the Si layer thickness is tuned from 10 to 200 nm (Figure 2(b)). The thickness of the Si layer is the key to tuning the color of the appearance as shown in the calculated color gamut in Figure 2(c) (Si thickness: 10–200 nm). The roughness of the substrate is controlled so that it scatters incident IR and visible lights, but it works like a smooth surface for incident microwave.

*Design of the MW scattering surface*: The MW scattering surface in the hierarchical design is a checkerboard structure that scatters the incident microwave based on phase cancellation. The designed MW scattering surface is shown in Figure 2(d). It consists of Al squares at two different heights (the width of each unit is 4 cm in this design). The reflection phase difference between the two adjacent units must be within the range of 180° ± 37° to maintain the RCSR under −10 dB [58]. The height difference results in different reflection phases between adjacent units (i.e., units A and B in Figure 2(d)). The relationship between the reflection phase difference and the height difference at the microwave frequency range (8–12 GHz, X band) is calculated and shown in Figure 2(e). The colormap in Figure 2(e) shows that when the height difference between unit A and unit B is 7.4 mm, the reflection phase difference can be kept within 180° ± 37° over the whole X band. For the MW scattering surface whose height difference is 7.4 mm, the simulated result of RCSR over the whole X band is under −10 dB (Figure 2(f)), which originates from the scattering of the incident microwave (see the far-field patterns in Figure S2, Section 3 of Supplementary Material).

The underlying physical mechanisms behind color tunability, VIS-IR scattering, and MW scattering are different for the hierarchical VIS-IR-MW scattering surface. The color tunability can be explained by the multiple reflection of light within the thin Si layer and their interference [59] (Figure S3); the VIS-IR scattering happens due to the geometrical reflection of light from the rough surface (Figure S4); and the MW scattering occurs due to the diffraction of the incident microwave, which can be explained using Fourier optics [60] (Figure S5).

### 3.2 Experimental results

#### 3.2.1 Characterization of the fabricated samples

The hierarchical VIS-IR-MW scattering surface and a low-emissivity smooth checkerboard (for reference) are fabricated and characterized (Figure 3). Both devices have Al surfaces and are arranged as 4 × 4 arrays (each unit square is 4 × 4 cm, and the total size is 16 × 16 cm). The visible images are shown in Figure 3(a). The hierarchical VIS-IR-MW scattering surface is rough (the bulges on the surface are of several tens of micrometers) compared to the low-emissivity smooth checkerboard, as shown in the scanning electron microscope (SEM) images in Figure 3(a).

*IR emissivity and scattering property*: The measured average emissivities at the 8–14 μm wavelength range for the hierarchical VIS-IR-MW scattering surface and the low-emissivity smooth checkerboard are 0.17 and 0.1, respectively (Figure 3(b)). Both devices present low IR emissivity, ensuring the camouflaged target emits low IR radiation so that it is not exposed to an IR camera. The average specular reflectivity of the hierarchical VIS-IR-MW scattering surface is 0.13 over 8–14 μm. However, for the low-emissivity smooth checkerboard, it comes out to be 0.97, which is 7.5 times higher than the hierarchical VIS-IR-MW scattering surface (see Figure 3(c)). Since both devices present low IR emissivity, the lower specular reflection indicates the higher scattering. Thus, the hierarchical VIS-IR-MW scattering surface has a much more robust capability to scatter the incident IR light and overcome the influence of an external heat source, compared to the low-emissivity smooth MW scattering checkerboard. Furthermore, based on the morphology of the VIS-IR scattering surface (Figure 3(g)), statistical results of scattering intensity versus altitude angel *θ* are calculated (blue bar in Figure 3(h)), which fit well with that of an ideal Lambertian surface (orange line in Figure 3(h)), suggesting the nearly perfect scattering property of the VIS-IR scattering surface (for detailed analysis, please see the physical models in Section 4 of Supplementary Material).

*RCSR in microwave range*: RCSR is measured to characterize the capability of MW camouflage for the hierarchical VIS-IR-MW scattering surface and the low-emissivity smooth checkerboard, respectively (Figure 3(d)). Both devices can maintain the RCSR below −10 dB over 8–13 GHz, suggesting that the VIS-IR scattering surface in the hierarchical design does not influence the property over microwave range, and both devices can realize good MW camouflage over the whole X band (8–12 GHz).

*VIS camouflage and color tunability*: VIS camouflage is obtained by covering a Si layer of different thicknesses onto an Al layer of the VIS-IR scattering surface of the hierarchical design. The VIS-IR scattering surface consisting of a 20 nm-thick Si top layer can mimic the appearance of a leaf exceedingly well, presenting the capability of VIS camouflage (see the top panel in Figure 3(e)). The color of the VIS-IR scattering surface can be controlled by changing the thickness of the Si layer (the middle panel in Figure 3(e)). When heated at 100 °C, all the VIS-IR scattering surfaces with Si layers of different thicknesses can maintain low apparent temperature (the temperature displayed by the IR camera, *T*_IR_) in contrast to the high apparent temperature (close to 100 °C) of Polyimide (PI) film – high emissivity, near 1 (see the bottom panel of Figure 3(e)). This suggests that the Si layer hardly influences the IR camouflage performance of the VIS-IR scattering surface. The measured visible spectra (Figure 3(f)) of the VIS-IR scattering surfaces (with Si layers of 20, 70, and 110 nm thicknesses) show that the specular reflection presents a huge difference with the integral reflection (whole scattered energy in the hemispherical space), proving that the VIS-IR scattering surfaces also exhibit high scattering in VIS range.

#### 3.2.2 IR camouflage performance

**Figure 4:**
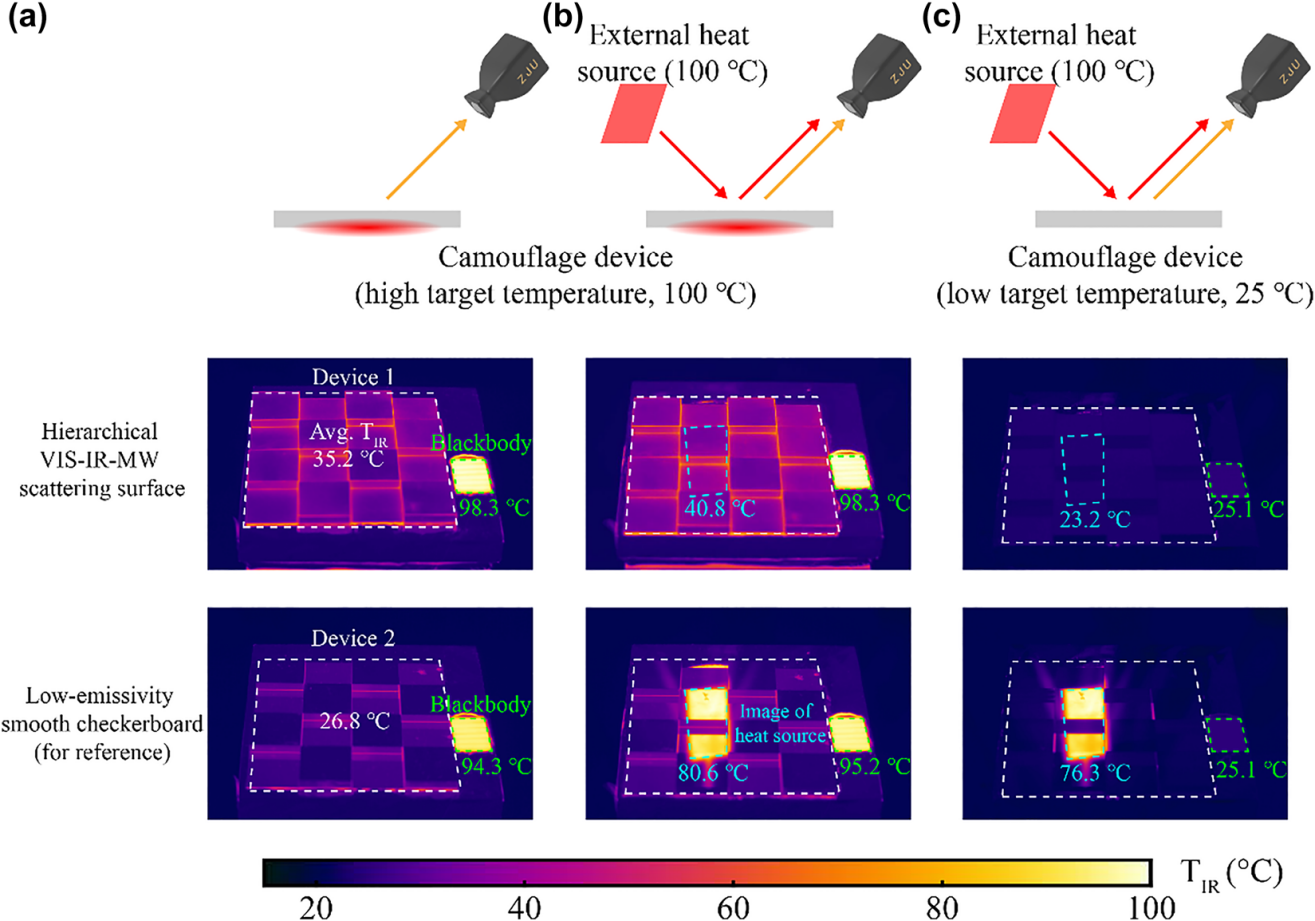
Experimental performance of IR camouflage. IR camouflage is performed in three scenarios: (a) high target temperature (100 °C) and without an external heat source (100 °C) nearby; (b) high target temperature (100 °C) and with an external heat source (100 °C) nearby; (c) low target temperature (25 °C) and with an external heat source (100 °C) nearby. The top panel presents the schemes of the three scenarios. The middle panel shows the IR images of the hierarchical VIS-IR-MW scattering surface. The bottom panel shows the IR images of the low-emissivity smooth checkerboard for reference. The average apparent temperature of the dashed box regions is shown in the IR images (white: device, green: blackbody, cyan: the image of the external heat source).

The IR camouflage performance of the hierarchical VIS-IR-MW scattering surface is tested under an IR camera, while the low-emissivity smooth checkerboard is used as a reference (Figure 4).

In the IR camouflage scenario without an external heat source, the IR signal detected by the IR camera mainly comes from the IR radiation of the devices. When camouflaging a target of 100 °C, the hierarchical VIS-IR-MW scattering surface presents a much lower apparent temperature (average 35.2 °C) compared to the blackbody (average 98.3 °C) (Figure 4(a)), indicating that the radiation intensity is reduced by 54%. It implies that the hierarchical VIS-IR-MW scattering surface can effectively reduce the IR radiation from the camouflaged target.

In the IR camouflage scenario with an external heat source, the IR signal detected by the IR camera comes not only from the IR radiation of the devices but also from the reflection of the external heat source. When camouflaging a target of high/low temperature (100 °C/25 °C) and with an external heat source (100 °C) nearby, the hierarchical VIS-IR-MW scattering surface presents excellent capability to overcome the influence of the external heat source in contrast to the low-emissivity smooth checkerboard. For the hierarchical VIS-IR-MW scattering surface, the reflected image region of the external heat source (cyan dashed box in the IR images, Figure 4) and the non-reflected image region show hardly any difference in apparent temperature (middle panels in Figure 4(b) and (c)). However, for the low-emissivity smooth checkerboard, the reflected image region presents a much higher apparent temperature than the non-reflected image region (bottom panels in Figure 4(b) and (c)). In terms of the reflected image region of the external heat source, when camouflaging a target of 100 °C (25 °C), the hierarchical VIS-IR-MW scattering surface presents an average apparent temperature of 40.8 °C (23.2 °C), while the low-emissivity smooth checkerboard presents 80.6 °C (76.3 °C); for the hierarchical VIS-IR-MW scattering surface, the radiation intensity from the reflected image region is 39% (51%) less compared to the low-emissivity smooth checkerboard.

Moreover, the hierarchical VIS-IR-MW scattering surface presents excellent IR camouflage performance against the change of target temperature (20–100 °C), the temperature of the external heat source (40–100 °C), and the position of the external heat source (the incident angle is changed over 30°) – shown in Section 5 of Supplementary Material.

### 3.3 Analysis of the effect of MW scattering on IR camouflage

MW scattering camouflage can reduce the device’s temperature and thereby help improve the IR camouflage capability, unlike the MW absorption camouflage (see Section 6 of Supplementary Material). Heat transfer with incident microwave power is simulated for two similar checkerboard structures – one with an MW scattering surface and the other with an MW absorption surface. The average temperature of the checkerboard is calculated for both cases and shown in Figure S9. When the incident microwave power is 1000 W/m^2^ (equivalent to the power at a distance of 2.2 km to a sea-based X band radar with a transmitter power output of 20 kW and a gain of 65 dB), it turns out that the device temperature of the MW scattering surface (52.4 °C) is much less compared to the MW absorption surface (94.8 °C), which is beneficial to the IR camouflage.

## 4 Conclusions

We demonstrate a hierarchical VIS-IR-MW multispectral scattering camouflage device that reduces heat production from microwave absorption and improves its capability to overcome the influence of external heat sources in contrast to reported multispectral camouflage works (see Table S1 in Section 7 of the Supplementary Material for the comparison of multispectral camouflage performances between this work and prior works). First, besides the visible, LWIR, and microwave bands, the demonstrated hierarchical VIS-IR-MW scattering surface in this work also exhibits low specular reflection in the near-infrared (0.75–1.4 μm) and the short-wavelength infrared (1.4–3 μm) ranges (see the measured specular reflection spectrum of the VIS-IR scattering surface, Figure S10, Section 8 of Supplementary Material) enabling the device to be camouflaged from laser radar (e.g., 905 and 1550 nm laser) over a broad wavelength range. Second, the demonstrated hierarchical VIS-IR-MW scattering surface not only hides the IR signal of the target behind it but also conceals the IR signal of the nearby objects (e.g., an external heat source of 100 °C) by reducing the specular reflection. Third, the −10 dB RCSR bandwidth can be further expanded to improve the MW camouflage by tuning the arrangement of the structural units (referring to the principle of coding metasurfaces) and adding more structure units with different reflection phases. Fourth, the metal in the hierarchical VIS-IR-MW scattering surface can be replaced by transparent conducting materials (e.g., ITO) to make the entire device transparent and expand the application scenarios. Fifth, phase change materials (e.g., WO3, GST and VO2 [61, 62]) can be utilized to realize dynamic emission and reflection control. Lastly, the hierarchical VIS-IR-MW scattering surface can be intrinsically mass-produced and can serve as a platform for potentially facilitating applications in optical deception, energy generation, and thermal management [63–67].

## Supplementary Material

Supplementary Material Details
