# Supplementary material for: Hierarchical visible-infrared-microwave scattering surfaces for multispectral camouflage
Source: Nanophotonics. 2022 Jul 14;11(16):3613–22. doi: 10.1515/nanoph-2022-0254 (PMC11501711; doi:10.1515/nanoph-2022-0254)
Supplement: Supplementary file 1 — Supplementary Material Details [file j_nanoph-2022-0254_suppl.doc]

Supporting Information

1. Calculation of the spectrum of the Si-Al layered structure

Transfer-matrix method (TMM) is utilized to calculate the spectrum of the Si-Al layered structure. The schematic of TMM is demonstrated in Fig. S1. The multilayer system (Fig. S1) consists of n layers. Layer *i* has dielectric constant *𝜀_i_* and thickness *d_i_*. *z_i_* represents the interface between layer *i* and layer *i+1*. *a^i^_+_* and *a^i^-* are the forward and backward propagating wave inside layer *i*. Here, we assume that *a^i^_+_* and *a^i^-* are both at the left side of the interface *z_i_*. *r* and *t* are reflection coefficient and transmission coefficient, respectively.


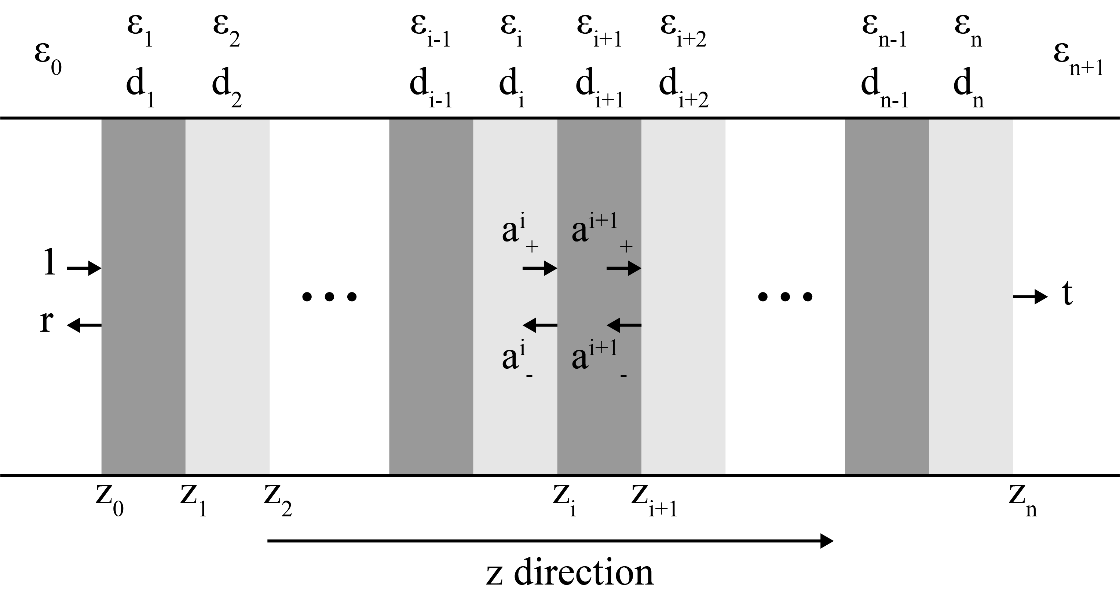


**Fig. S1:** Schematic of TMM.

In this work, we only consider the condition of normal incidence since the spectrum of layered structure is hardly influenced by the incident angle. According to boundary condition of electromagnetic fields, (*a^i^_+_*, *a^i^-*) and (*a^i+1^_+_*, *a^i+1^-*) can be related by Equation (S1) and Equation (S2):

$$\text{a}_{+}^{i}+a_{-}^{i}=a_{+}^{i+1}e^{-jk_{z}^{i+1}d_{i+1}}+a_{-}^{i+1}e^{jk_{z}^{i+1}d_{i+1}}$$

$$\sqrt{\varepsilon_{i}}\text{a}_{+}^{i}-\sqrt{\varepsilon_{i}}a_{-}^{i}=\sqrt{\varepsilon_{i+1}}a_{+}^{i+1}e^{-jk_{z}^{i+1}d_{i+1}}-\sqrt{\varepsilon_{i+1}}a_{-}^{i+1}e^{jk_{z}^{i+1}d_{i+1}}$$

where *k^i^_z_* represents the propagation constant along *z* direction in layer *i*.

Combining Equation (S1) and Equation (S2), the following matrix equation can be derived:

$$\left[\begin{matrix} a_{+}^{i} \\ a_{-}^{i} \end{matrix}\right]=\left[\begin{matrix} \frac{1}{2}\left(1+\sqrt{\frac{\varepsilon_{i+1}}{\varepsilon_{i}}}\right)e^{-jk_{z}^{i+1}d_{i+1}} & \frac{1}{2}\left(1-\sqrt{\frac{\varepsilon_{i+1}}{\varepsilon_{i}}}\right)e^{jk_{z}^{i+1}d_{i+1}} \\ \frac{1}{2}\left(1-\sqrt{\frac{\varepsilon_{i+1}}{\varepsilon_{i}}}\right)e^{-jk_{z}^{i+1}d_{i+1}} & \frac{1}{2}\left(1+\sqrt{\frac{\varepsilon_{i+1}}{\varepsilon_{i}}}\right)e^{jk_{z}^{i+1}d_{i+1}} \end{matrix}\right]\left[\begin{matrix} a_{+}^{i+1} \\ a_{-}^{i+1} \end{matrix}\right]$$

For simplicity, the Equation (S3) can be written as:

$$\left[\begin{matrix} a_{+}^{i} \\ a_{-}^{i} \end{matrix}\right]=M_{i}\left[\begin{matrix} a_{+}^{i+1} \\ a_{-}^{i+1} \end{matrix}\right]$$

Then, the relation between (*1*, *r*) and (*a^n^_+_*, *a^n^-*) can be derived as:

Here,

$$M_{i}=\left[\begin{matrix} \frac{1}{2}\left(1+\sqrt{\frac{\varepsilon_{i+1}}{\varepsilon_{i}}}\right)e^{-jk_{z}^{i+1}d_{i+1}} & \frac{1}{2}\left(1-\sqrt{\frac{\varepsilon_{i+1}}{\varepsilon_{i}}}\right)e^{jk_{z}^{i+1}d_{i+1}} \\ \frac{1}{2}\left(1-\sqrt{\frac{\varepsilon_{i+1}}{\varepsilon_{i}}}\right)e^{-jk_{z}^{i+1}d_{i+1}} & \frac{1}{2}\left(1+\sqrt{\frac{\varepsilon_{i+1}}{\varepsilon_{i}}}\right)e^{jk_{z}^{i+1}d_{i+1}} \end{matrix}\right]$$

At the interface *z_n_*, (*a^n^_+_*, *a^n^-*) and *t* can be related by boundary condition of electromagnetic fields,

$$\left[\begin{matrix} a_{+}^{n} \\ a_{-}^{n} \end{matrix}\right]=P\left[\begin{matrix} t \\ 0 \end{matrix}\right]$$

Here,

$$P=\left[\begin{matrix} \frac{1}{2}\left(1+\sqrt{\frac{\varepsilon_{n+1}}{\varepsilon_{n}}}\right) & \frac{1}{2}\left(1-\sqrt{\frac{\varepsilon_{n+1}}{\varepsilon_{n}}}\right) \\ \frac{1}{2}\left(1-\sqrt{\frac{\varepsilon_{n+1}}{\varepsilon_{n}}}\right) & \frac{1}{2}\left(1+\sqrt{\frac{\varepsilon_{n+1}}{\varepsilon_{n}}}\right) \end{matrix}\right]$$

Combining Equation (S5) and Equation (S7), we can get:

$$\left[\begin{matrix} 1 \\ r \end{matrix}\right]=M\left[\begin{matrix} t \\ 0 \end{matrix}\right]$$

Here,

Finally, the reflectivity R can be calculated by:

$$R=\left|r\right|^{2}=\left|\frac{M_{21}}{M_{11}}\right|^{2}$$

By substituting the parameters of Si and Al, the reflection spectrum of the Si-Al layered structure can be calculated.

1. Calculation of the color gamut

Based on the calculated reflectivity ¬of the Si-Al layered structure and under AM1.5 solar irradiance, the tristimulus values (X, Y, Z) in the CIE 1931 system can be calculated by the following formulas:

$$\text{X}=\int_{360nm}^{800nm} \frac{f_{x}(\lambda)R(\lambda)I(\lambda)}{4\pi}d\lambda$$

$$Y=\int_{360nm}^{800nm} \frac{f_{y}(\lambda)R(\lambda)I(\lambda)}{4\pi}d\lambda$$

$$Z=\int_{360nm}^{800nm} \frac{f_{z}(\lambda)R(\lambda)I(\lambda)}{4\pi}d\lambda$$

Here, *f_x_*, *f_y_* and *f_z_* are the stimulus functions in the CIE 1931 system. *R* is the reflectivity. *I* represents the solar irradiance. The calculation is performed over the spectrum range of 360-800 nm.

Then the chromaticity coordinate (*x*, *y*) can be calculated by:

$$\text{x}=\frac{X}{X+Y+Z}$$

$$\text{y}=\frac{Y}{X+Y+Z}$$

For Si-Al layered structures with Si layer of different thickness, the chromaticity coordinates are shown in Fig. 2c.

1. Simulation of the MW property of the checkerboard structure

The checkerboard structure of 4 x 4 arrays (as Fig. 2d shows) is built in commercial software Lumerical FDTD to study the MW property. Normal direction is set for the incident microwave in the simulation. The simulated far field patterns of the checkerboard are presented in Fig. S2, showing high scattering at 9 GHz, 10 GHz and 11 GHz. RCSR is calculated by dividing the normally reflected energy flux of the checkerboard to that of an Al plane (of the same size). The calculated RCSR is shown in Fig. 2f. Since every single unit in the checkerboard is an Al plane, which has high reflection (near 1) over MW range, the RCSR of the checkerboard structure mainly results from scattering.


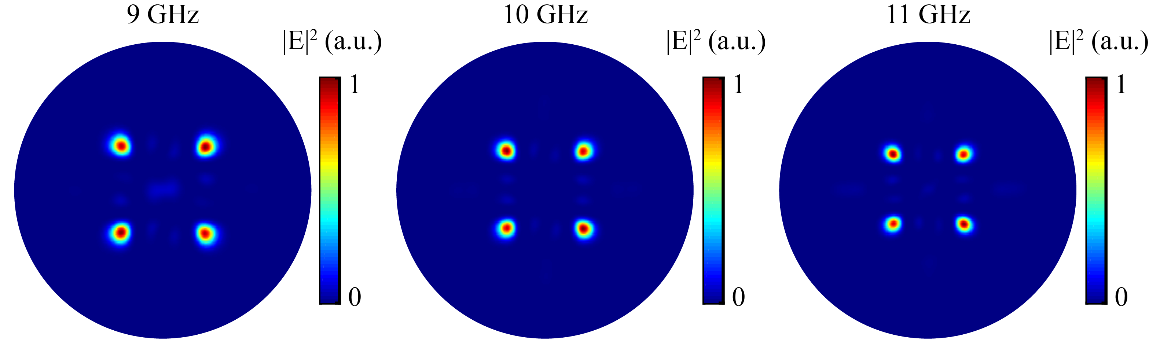


**Fig. S2:** Far field patterns (at 9 GHz, 10 GHz and 11 GHz) of the checkerboard structure.

1. Physical models for the properties in VIS, IR, and MW range

The properties of the hierarchical VIS-IR-MW scattering surface in the three different spectrum ranges are explained by i) the multiple reflection and interference of light in the Si layer for color tunability, ii) geometrical reflection of light from the rough surface for VIS-IR scattering, and iii) Fourier optics for MW scattering.

*Color tunability*: The color tunability in VIS range can be explained by the multiple reflection and interference of light (multiple reflection model) (Fig. S3(a)). The total reflection can be interpreted as the superposition of the partial waves reflected from the top and bottom surfaces of the Si layer. Consider the corresponding reflection coefficients to be *r_0_*, *r_1_*, *r_2_*, *r_3_*, and so on (Fig. S3(a)). The total reflectivity *R* can be written as

$$\text{R=}\left|\sum_{n=0}^{\infty} r_{n}\right|^{2}$$

Here, *r_n_* represents the reflection coefficient of the n^th^ reflected wave.

Specifically,

$$\text{r}_{0}=r_{12}$$

The general expression for *r_n_* can be given by

$$r_{n}=t_{12}r_{23}(r_{21}r_{23})^{n-1}t_{21}e^{i*2n\beta}$$

Here, *r_pq_* and *t_pq_* are the Fresnel reflection and transmission coefficients as the wave encounters medium *q* from medium *p*. For normal incidence,

$r_{pq}=\frac{\mathop{n}\limits^{\sim} _{p}-\mathop{n}\limits^{\sim} _{q}}{\mathop{n}\limits^{\sim} _{p}+\mathop{n}\limits^{\sim} _{q}}$,

$t_{pq}=\frac{2\mathop{n}\limits^{\sim} _{p}}{\mathop{n}\limits^{\sim} _{p}+\mathop{n}\limits^{\sim} _{q}}$, and

$$\beta=\frac{2\pi}{\lambda}\mathop{n}\limits^{\sim} _{2}h$$

where $\mathop{n}\limits^{\sim} _{1}$, $\mathop{n}\limits^{\sim} _{2}$ and $\mathop{n}\limits^{\sim} _{3}$ are the complex refractive indices of air, Si and Al, respectively. *λ* is the wavelength, and *h* is the thickness of the Si layer.

Since Si is a lossy material in VIS range, finite number of partial waves is enough to calculate the total reflectivity. As Fig. S3(b) shows, the reflectivity calculated from the multiple reflection model (using 10 partial waves, *r_0_* –*r_9_*, red dotted line) fits well with that calculated from TMM (blue line).


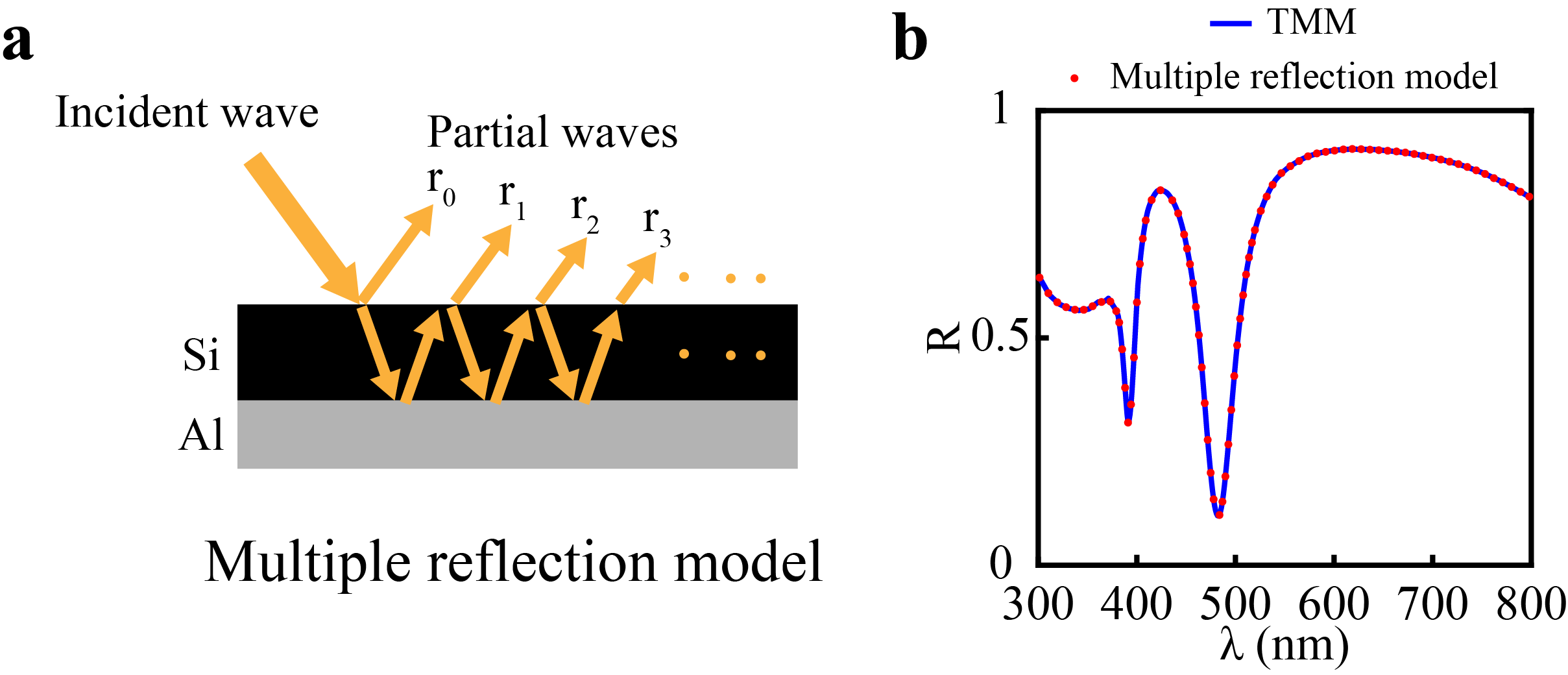


**Fig. S3:** (a) Schematic of multiple reflection model. (b) Calculated reflectivity from TMM (blue line) and the multiple reflection model (red dotted line).

*VIS-IR scattering*: Since the sizes of the particles and the distance between them (~100 μm) are much larger than the incident VIS (380–780 nm) and IR (8–14 μm) light wavelength (Fig. 3a), geometrical optics is suitable to describe the scattering property of the VIS-IR scattering surface. The morphology of the VIS-IR scattering surface is measured by a white light optical profiler (NT9100) (right panel of Fig. S4(a)). Normal incidence is assumed and specular reflections are calculated for each face element of the VIS-IR scattering surface (left panel of Fig. S4(a)). The reflections along the altitude angle *θ* (from 0° to 89°, with interval of 1°; the reflections between face elements are considered) are counted and considered as the scattering intensity of *θ* (i.e., the scattering within the ring part in the left panel of Fig. S4(b)). The statistical calculation results (blue bars) of the intensity of *θ* are shown in Fig. S4(b), which fit well with the scattering intensity of an ideal Lambertian surface ($\sim\sin\theta\cos\theta$, orange line), suggesting the nearly perfect scattering property of the VIS-IR scattering surface.


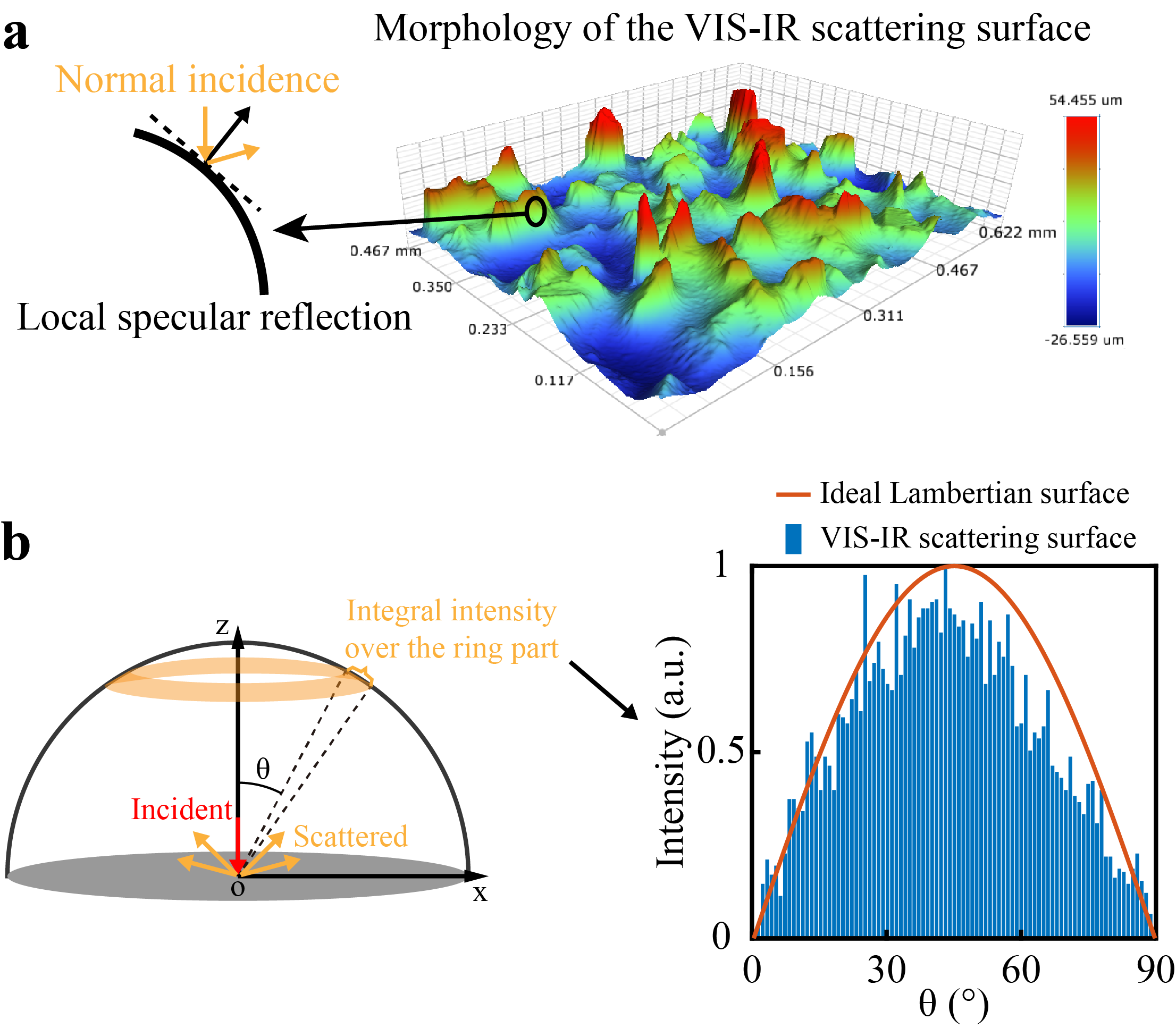


**Fig. S4:** (a) The morphology of the VIS-IR scattering surface and the scheme of the local specular reflection. (b) Scattering intensity (integral in the orange ring part, left panel) versus the altitude angel *θ* of the VIS-IR scattering surface (blue bar, statistical calculation results based on the morphology data in (a)) and ideal Lambertian surface (orange line). For the statistical calculation results, the scattering intensity along each *θ* is counted from 0° to 89°, with an interval of 1°.

*MW scattering*: The unit length (4 cm) of the MW scattering surface (checkerboard structure) is close to the incident microwave wavelength (e.g., 10 GHz ~ 3 cm). The scattering in the MW range originates from the diffraction of the incident microwave, which can be interpreted by Fourier optics. In the case where the phase difference of two adjacent units in the MW scattering surface is π, the diffraction of the incident microwave can be interpreted as the interference of multiple propagating plane waves along different direction, which interfere on the plane (the original plane) and form the same pattern of electric field (4 x 4 square arrays; unity magnitude and the same phase in each square; adjacent squares have phase difference of π) (Fig. S5(a)). After passing the plane, these waves continue to propagate and form the diffraction pattern. For far-field diffraction (or Fraunhofer diffraction), the intensity of each direction (for a specific altitude angle *θ* and a specific azimuth angle *φ*) is proportional to that of a decoupled plane wave along the same direction, which can be derived from Fourier transform of the electric field pattern on the original plane, i.e.,

$$I(\theta,\varphi)\propto\left|\int_{-\infty}^{\infty} \int_{-\infty}^{\infty} E(x,y)e^{ik\sin\theta(x\cos\varphi+y\sin\varphi)}dxdy\right|^{2}$$

Here, *E(x,y)* represents the electric field distribution on the original plane, *k* represents the wavenumber of the microwave.

Using this formula, the far-field intensity distribution can be calculated for the MW scattering surface (left panel of Fig. S5(b)), which fits well with the simulated result in FDTD (right panel of Fig. S5(b)).


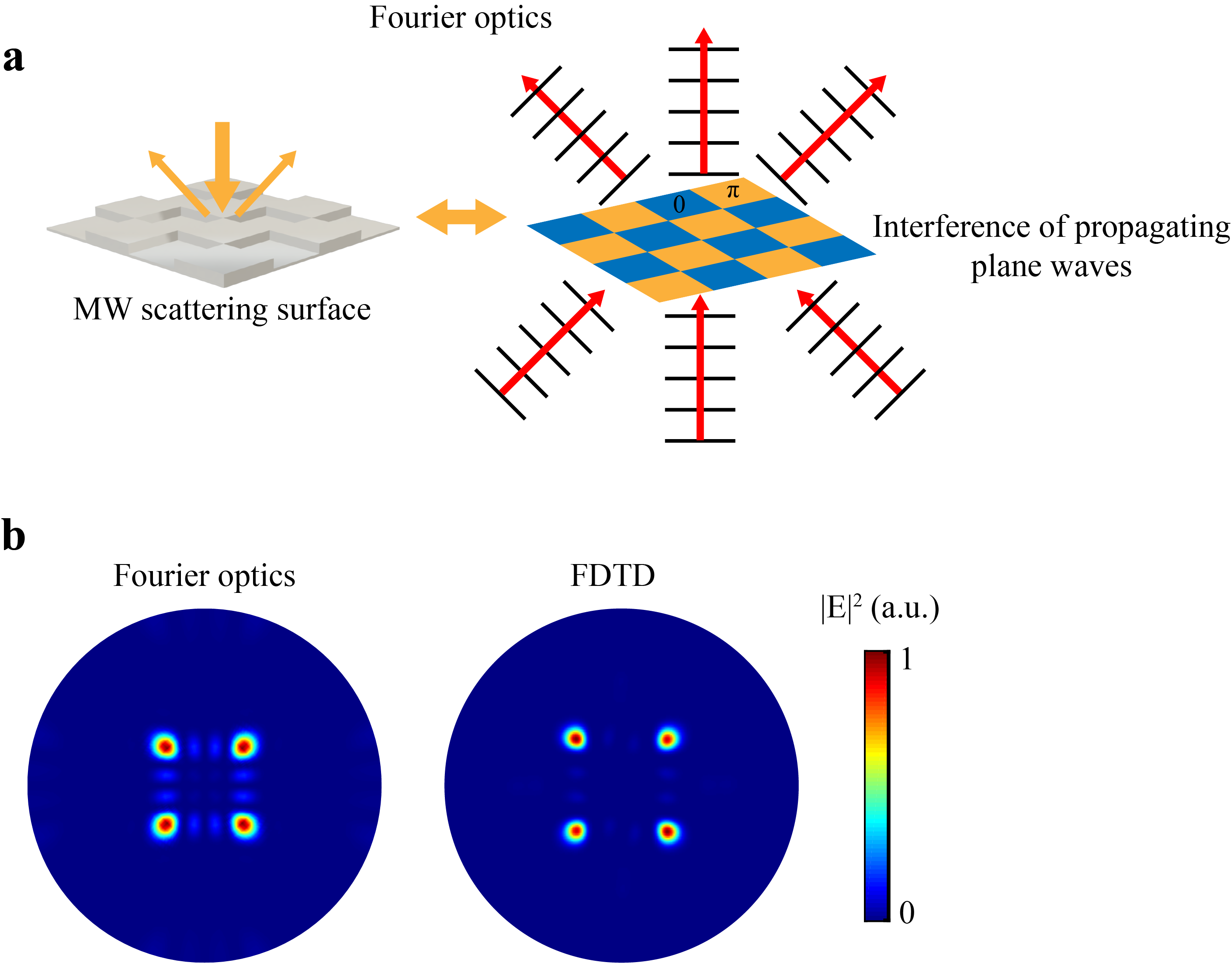


**Fig. S5:** (a) Schematic of Fourier optics. (b) The calculated far-field intensity distribution by Fourier optics (left) and FDTD (right).

1. Factors influencing IR camouflage performance

The IR camouflage performance of the hierarchical VIS-IR-MW scattering surface is studied in terms of the target temperature, the temperature of the external heat source and the position of the external heat source.

*The influence of the target temperature*: The apparent temperature (displayed by the IR camera) is almost linear to the target temperature, and during increasing the target temperature, the hierarchical VIS-IR-MW scattering surface manifests the lower apparent temperature than the blackbody, as shown in Fig. S6(b). The growth rates of the average apparent temperature (the entire device) with the target temperature are 0.21 and 0.99 for the hierarchical VIS-IR-MW scattering surface and the blackbody, respectively. It suggests that for the hierarchical VIS-IR-MW scattering surface, the capability to overcome the influence of the target temperature is 4.7 times better than the blackbody.


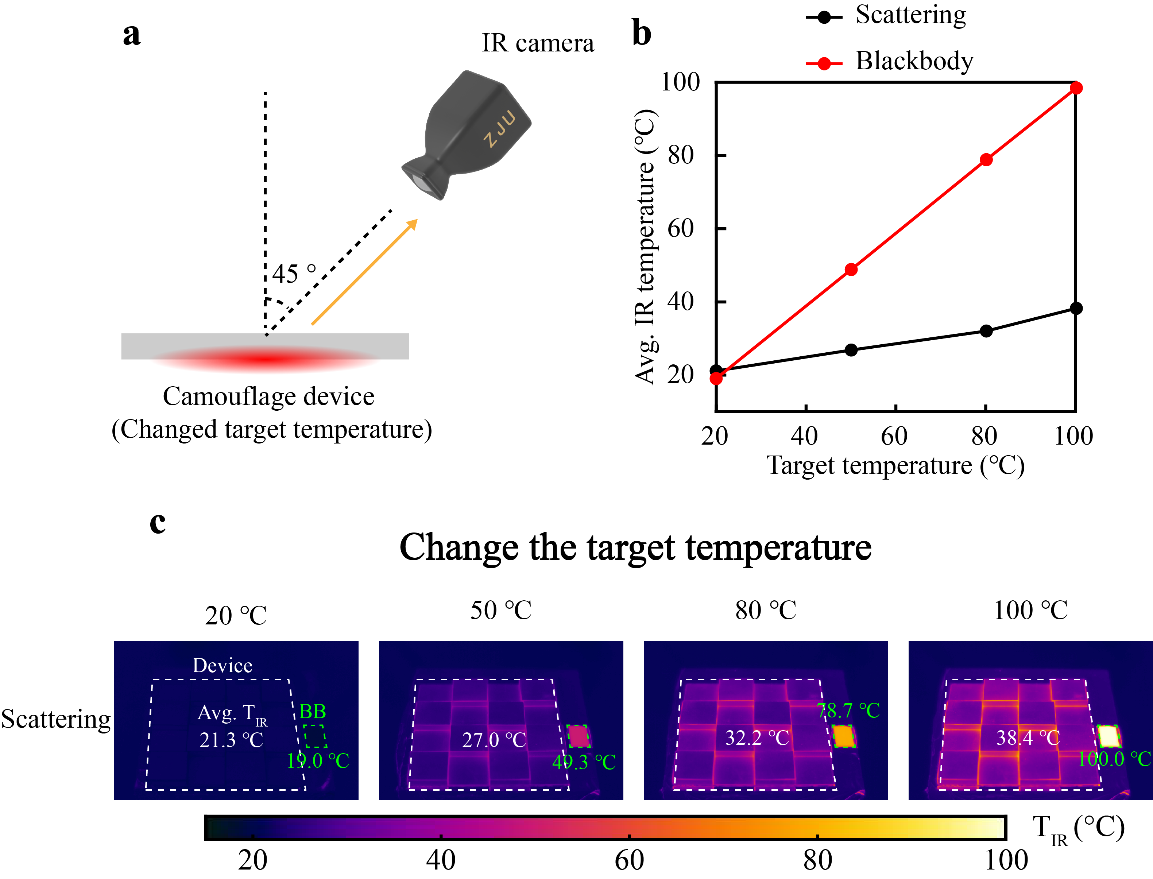


**Fig. S6:** The influence of target temperature. (a) The schematic of the test for the influence of target temperature on IR performance. (b) The relation between the average apparent temperature and the target temperature (black: the hierarchical VIS-IR-MW scattering surface, red: the blackbody). (c) IR images of the hierarchical VIS-IR-MW scattering surface under different target temperature. (white: the device region, green: the blackbody region)

*The influence of the temperature of the external heat source*: The apparent temperature is almost linear to the temperature of the external heat source, as Fig. S7(b) shows. The growth rates of the average apparent temperature of the reflected IR image region with the temperature of the external heat source are 0.08 and 0.60 for the hierarchical VIS-IR-MW scattering surface and the low-emissivity smooth checkerboard respectively (Fig. S7(b)). It suggests that for the hierarchical VIS-IR-MW scattering surface, the capability to overcome the influence of the temperature of the external heat source is 7.5 times better than that of the low-emissivity smooth checkerboard.


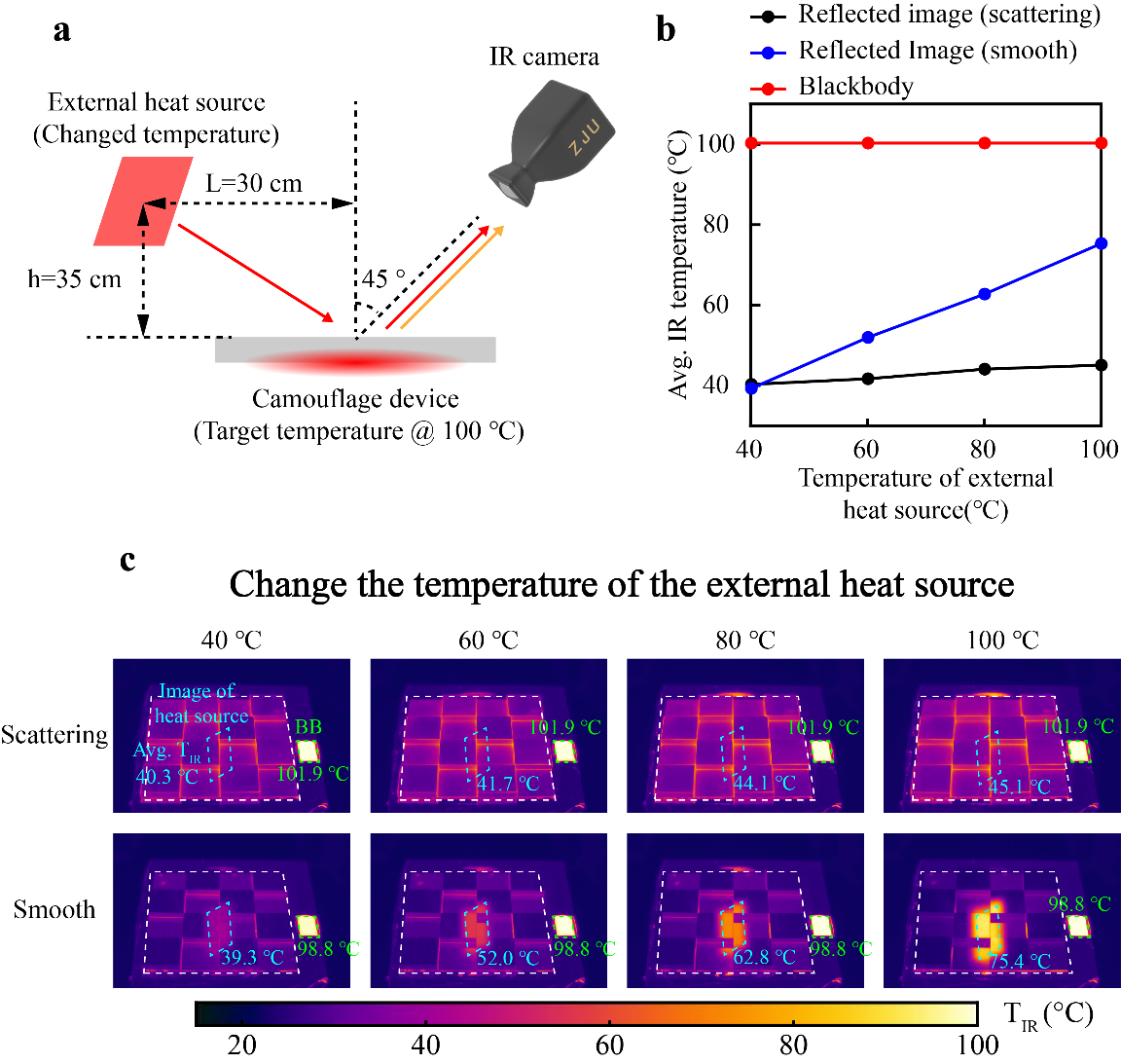


**Fig. S7:** The influence of the temperature of the external heat source. (a) The schematic of the test for the influence of the temperature of the external heat source on IR performance. (b) The relation between the average apparent temperature and the temperature of the external heat source (black: reflected IR image region on the hierarchical VIS-IR-MW scattering surface, blue: reflected IR image region on the low-emissivity smooth checkerboard, red: the blackbody). (c) IR images of the hierarchical VIS-IR-MW scattering surface (upper) and low-emissivity smooth checkerboard (down) with different temperature of the external heat source. (white: the device region, green: the blackbody region, cyan: reflected IR image region)

*The influence of the position of the external heat source*: The reflected IR image of the external heat source on the low-emissivity smooth checkerboard is hotter than that on the hierarchical VIS-IR-MW scattering surface despite the change of the position of the external heat source, as Fig. S8(c) shows. From the experimental result, the external heat source can influence the IR camouflage over a range of about 30 °.


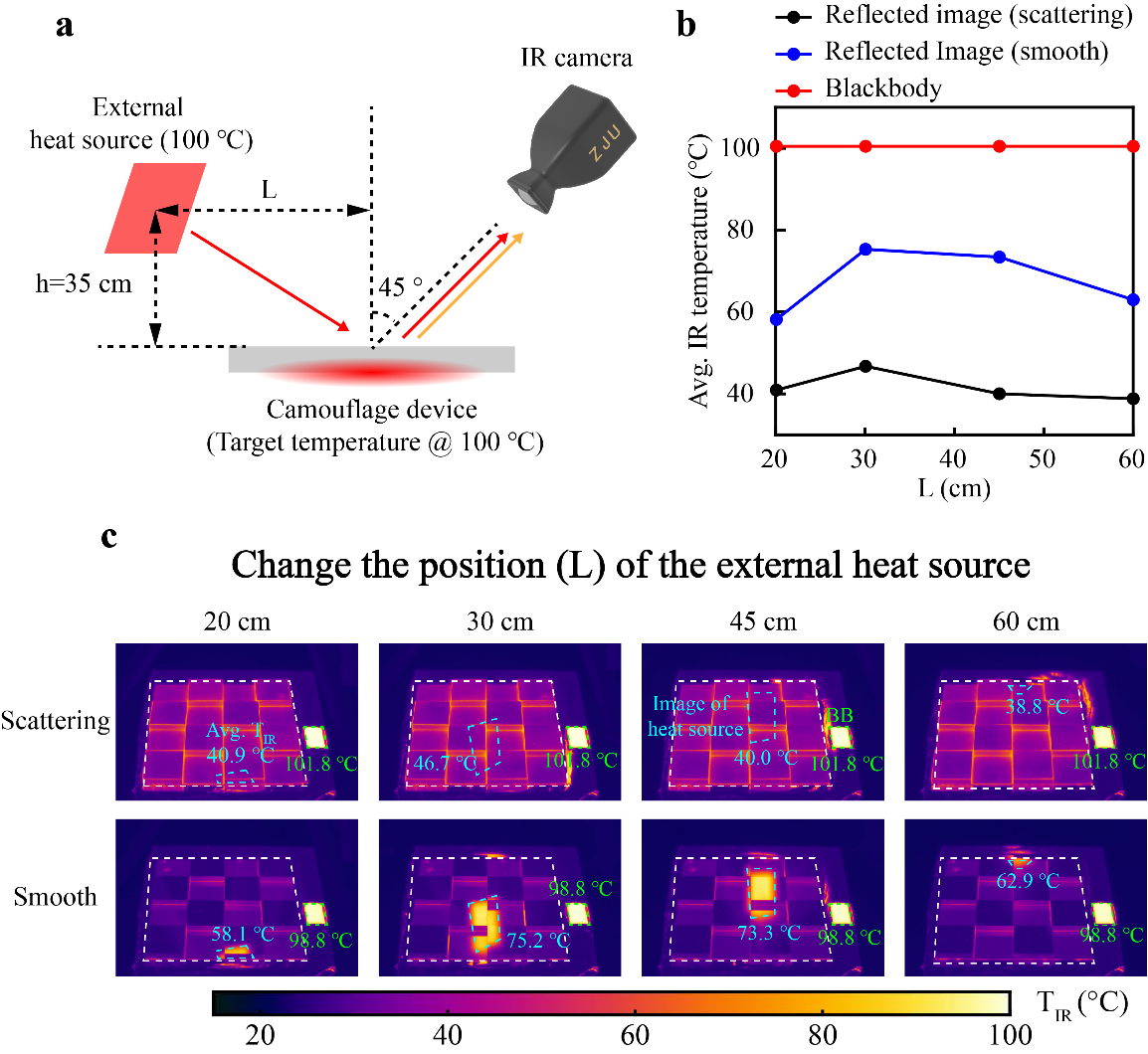


**Fig. S8:** The influence of the position of the external heat source. (a) The schematic of the test for the influence of the position of the external heat source on IR performance. (b) The relation between the average apparent temperature and the position of the external heat source (black: reflected IR image region on the hierarchical VIS-IR-MW scattering surface, blue: reflected IR image region on the low-emissivity smooth checkerboard, red: the blackbody). (c) IR images of the hierarchical VIS-IR-MW scattering surface (upper) and low-emissivity smooth checkerboard (down) with different position of the external heat source. (white: the device region, green: the blackbody region, cyan: reflected IR image region)

1. Comparison between MW scattering camouflage and MW absorption camouflage

The influence of MW scattering camouflage on IR camouflage is studied. Two same models of Al checkerboard (one with a MW scattering surface, the other with a MW absorption surface) are built in Commercial software COMSOL. The input energy on the top surface refers to the absorbed microwave energy. The input energy from the bottom surface refers to the heat transferred from the target to be camouflaged (here this power is set as 1000 W/m^2^). For the MW scattering camouflage, the input energy on the top surface is set as 0 W/m^2^, while for the MW absorption camouflage the input energy is changed from 0 to 2000 W/m^2^. Assuming that the MW scattering camouflage and the MW absorption camouflage have the same RCSR as the measured result of the VIS-IR-MW scattering surface (Fig. 3d), the relation between the incident microwave energy and the average temperature of the checkerboard can be simulated (right panel in Fig. S9).


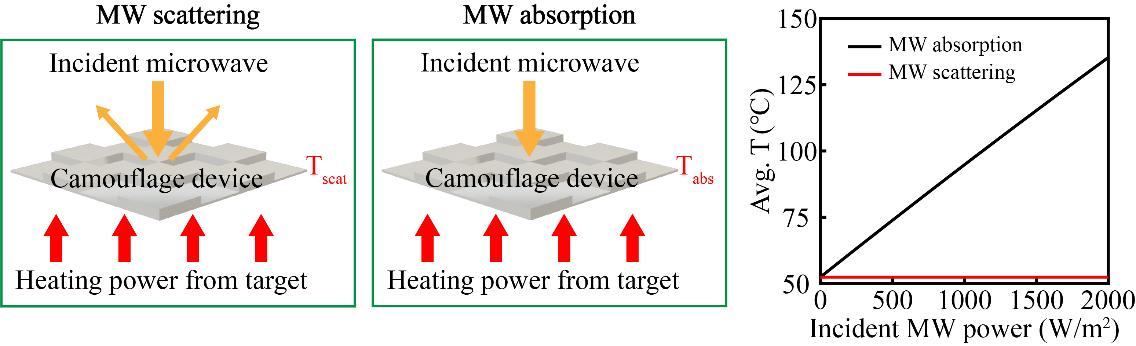


**Fig. S9:** Comparison between the influence of MW scattering and MW absorption on IR camouflage. Left: scheme for the simulation of the MW scattering checkerboard model. Middle: scheme for the simulation of the MW absorption checkerboard model. Right: simulated result of the relation between the average temperature of the checkerboard and the incident MW power (black: MW absorption, red: MW scattering).

1. Comparison of multispectral camouflage performances between this work and prior works

**Table 1: Comparison of multispectral camouflage performances between this work and prior works**

| Works | Method | VIS camouflage | IR camouflage | | MW camouflage | |
| --- | --- | --- | --- | --- | --- | --- |
|  |  |  | Low emission | High scattering | Absorption or  Transmission | Scattering |
| Ref. 14 | Dielectric-metal core-shell structure | Low reflection | Yes | Not reported | No | No |
| Ref. 17 | Graphene based device | Transmission tuning | Yes | No | Yes | No |
| Ref. 30 | Metasurface | No | Yes | Yes | No | No |
| Ref. 31 | Metasurface | Colorful | Yes | No | No | No |
| Ref. 42 | Photonic crystal | Colorful | Yes | No | Yes | No |
| Ref. 44 | Photonic crystal | Colorful | Yes | No | No | No |
| Ref. 45  (Simulation) | Metasurface | No | Yes | Yes | Yes | No |
| Ref. 46 | Metamaterial | Scattering | No | No | Yes | No |
| Ref. 47 | Photonic crystal && FSS* && Metasurface | Colorful | Yes | No | Yes | No |
| Ref. 48 | FSS && Metasurface | No | Yes | No | Yes | No |
| Ref. 50 | Metasurface | Colorful | Yes | No | Yes | No |
| Ref. 53 | FSS && Metasurface | No | Yes | No | Yes | No |
| This work | Hierarchical VIS-IR-MW scattering surfce | Colorful | Yes | Yes | No | Yes |

*FSS: Frequency selective surface

1. Measured specular reflection spectrum over near-infrared (0.75-1.4 μm) and short-wavelength infrared (1.4-3 μm) range

The specular reflectivity over near-infrared and short-wavelength infrared range is measured for the VIS-IR scattering surface (without Si) and a low-emissivity smooth surface (Al film on a Si substrate), as shown in Fig. S10. The VIS-IR scattering surface presents a much lower specular reflectivity (~0.1) than the low-emissivity smooth surface (~0.9) over the whole measured spectrum range.


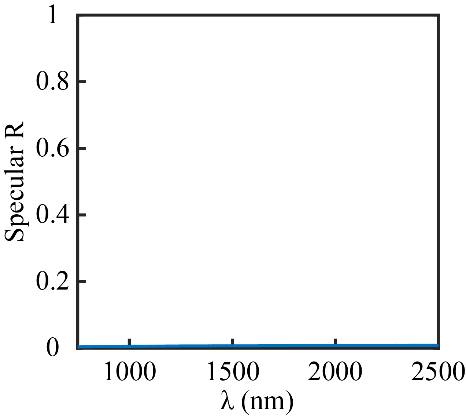


**Fig. S10:** The measured specular reflectivity over near-infrared and short-wavelength infrared range. (black: low-emissivity smooth surface, red: VIS-IR scattering surface)
